# Review of local injection of anti-TNF for perianal fistulising Crohn’s disease

**DOI:** 10.1007/s00384-017-2899-0

**Published:** 2017-09-12

**Authors:** Samuel O. Adegbola, Kapil Sahnan, Philip J Tozer, Robin KS Phillips, Omar D Faiz, Janindra Warusavitarne, Ailsa Hart

**Affiliations:** 1 Surgical Epidemiology, Trials and Outcome Centre (SETOC), St Mark’s Hospital and Academic Institute, Harrow, Middlesex, HA1 3UJ UK; 2 Department of Surgery, Imperial College, St Mary’s Hospital, Praed Street, London, W2 1NY UK; 3 St Mark’s Hospital, Watford Road, Harrow, HA1 3UJ UK; 4 Fistula Research Unit, St Mark’s Academic Institute, Harrow, Middlesex, HA1 3UJ UK

**Keywords:** Perianal, Anal, Crohn’s disease, Fistula, Medical, Surgical, Management, Biological therapies, anti-tumour necrosis factor-a

## Abstract

**Background:**

Perianal fistulising Crohn’s disease (PFCD) affects a third of Crohn’s disease patients and represents a disabling phenotype with poor outcome. The anti-tumour necrosis factor alpha (TNF) therapies have been shown to maintain clinical remission in a third of patients after 1 year of treatment. Maintenance therapy with systematic administration schedules confers greatest benefit, but exposes patients to risks/side effects of continued systemic use and led to consideration of local drug delivery (first described in 2000). In this review, we analyse all published articles on local anti-TNF therapy in the treatment of PFCD.

**Methods:**

The Preferred Reporting Items for Systematic Reviews and Meta-Analyses (PRISMA) guidelines were used to systematically search Medline and Embase using the medical subject headings ‘fistula’, ‘anus’, ‘Crohn disease’, ‘infliximab’ and ‘adalimumab’. This was combined with free text searches, e.g. ‘local injection’ and ‘Crohn’s perianal disease’. Studies/abstracts describing local injection treatment with anti-TNF were included in this review.

**Results:**

Six pilot studies including a total of 92 patients were included in this review. Outcomes reported were mostly clinical and included ‘complete/partial response’ to therapy and short-term results varied between 40 and 100%. There were no significant adverse events and the local injections were well tolerated.

**Conclusions:**

There is paucity of data assessing this treatment modality. Local anti-TNF therapy appears safe, but outcome reporting is heterogeneous, subjective and long-term data are unavailable. Our review suggests a potential role may be in those in whom systemic treatment is contraindicated and calls for standardised reporting of outcomes in this field to enable better data interpretation.

## Introduction

Perianal fistulising Crohn’s disease represents a particularly disabling phenotype of Crohn’s disease (CD) with poor outcomes. Incidence rises with increased duration of Crohn’s disease and reports of lifetime risk can be up to 40% [1]. It represents a distinct subset of Crohn’s disease as reflected in the Montreal classification of inflammatory bowel disease (IBD) [2] and often signifies an aggressive form of Crohn’s disease phenotype. Treatment of this condition has historically proved frustrating, often following a chronic and relapsing course, with up to 40% patients previously undergoing eventual proctectomy [3]. The advent of medical therapies, particularly biological therapy, heralded a positive change in the burden of disability associated with this condition. Anti-TNF therapy (i.e. infliximab, adalimumab) has been shown to maintain clinical remission in approximately a third of patients after 1 year of treatment [4]. Maintenance therapy with systematic administration schedules (rather than episodic use) of anti-TNF confers greatest benefit [5, 6]. However, this in turn exposes patients to the risks and side effects associated with continued use, including auto-antibody formation, infusion reactions, infections and malignancies [7]. This has led to consideration of local drug delivery, which was first described in 2000 [8]. In this review, we analyse all published articles on local anti-TNF therapy in the treatment of perianal fistulising CD.

## Methods

All articles/abstracts in the English literature reporting the use of local injection of anti-TNF for the treatment of perianal fistula in patients with Crohn’s disease were considered. The Preferred Reporting Items for Systematic Reviews and Meta-Analyses (PRISMA) guidelines were used to systematically search Medline and Embase (between January 2000 and December 2016) using the medical subject headings ‘fistula’, ‘anus’, ‘Crohn disease’, ‘infliximab’ and ‘adalimumab’. This was combined with free text searches, e.g. ‘local injection’, ‘Crohn’s perianal disease’ and cross-references. Studies/abstracts describing local injection treatment with anti-TNF-α were considered in this review. Retrieved citations and abstracts were reviewed by two independent reviewers (SOA and KS) and all relevant articles were selected. Any discrepancies in article selection were discussed and a final consensus was agreed. All relevant articles/abstracts describing the patient population with a sample size ≥ 5 patients were included. Data from the articles were expressed in spreadsheet format (using Microsoft Excel, Microsoft, Redmond, WA) and analysed to ascertain conclusions (where possible) from their collective information. Data extracted included participant numbers, age range, gender distribution, type/dose/dosing regimen of anti-TNF agent, duration of follow-up and endpoints, including complications. Quantitative data analysis was not possible due to methodological differences amongst the studies. Data were pooled without formal statistical analysis and meta-analysis due to study heterogeneity, small patient numbers and lack of comparative studies.

## Results

**Fig. 1:**
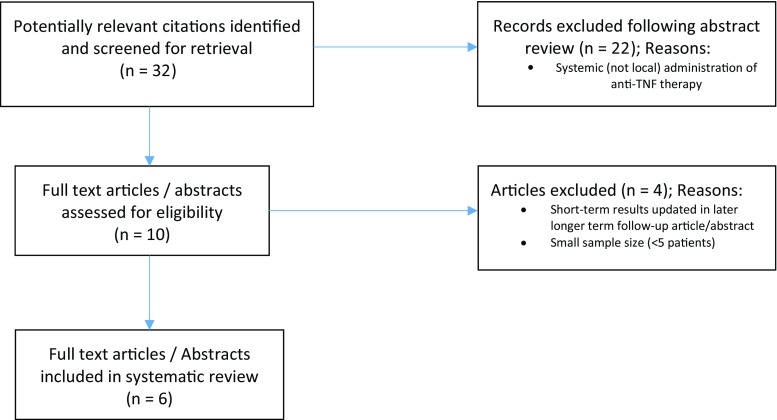
Flow diagram of search strategy

Six studies (four original articles, two cohort study abstracts) were included in this review [9–14] as demonstrated in the flowchart in Fig. 1 delete. A total of 92 patients were evaluated and the demographics are demonstrated in Table 1. All studies were pilot/prospective cohort studies. In all studies, patients largely continued concomitant therapy (i.e. steroids, 6-mercaptopurine, mesalazine, azathioprine). Tonelli et al. [13] stated that in their study, no patient received therapy with systemic immunosuppressive drugs. In the remaining five studies [9–12, 14], some included patients on concurrent systemic anti-TNF therapy [10], whereas the rest did not explicitly report this. Four studies [9–12] assessed infliximab as the local anti-TNF agent used, whilst the remaining two [13, 14] assessed adalimumab. Technique of injection, where specified, was similar between studies with examination under anaesthesia, curettage [13] of the fistula tract (in one case fistulectomy [10]) and injection of anti-TNF agent along the fistula tracts and circumferentially around the external and/or internal openings. Doses given varied between 15–25 mg infliximab and 20–40 mg adalimumab. Dosing intervals varied between 2 and 6 weeks. All treatment regimens employed by the varying studies required at least two sessions of treatment (i.e. injections at the varying time intervals, see Table 2). Follow-up ranged from 1 to 43 months.

**Table 1:** Results of demographics

Study	Design	Numbers	Age in years and median (range)	Male:female ratio	Type of fistula	Type of LA treatment
Lichtiger S. 2001 [9] (USA)	Pilot	9	NS	NS	NS	Infliximab
Poggioli et al. 2005 [12] (Ita)	Pilot	15	29.7	12:3	12 high TS 2 high IS 1 SS	Infliximab
Asteria et al. 2006 [11] (Ita)	Pilot	11	38.9 (28–44)	4:7	7 low TS 1 low IS 3 low AV/TS	Infliximab
Alessandroni et al. 2011 [10] (Ita)	Prospective cohort/pilot	12	40 (18–52)	8:4	5 high TS 5 TS 2 IS	Infliximab
Laureti et al. 2012 [14] (Ita)	Pilot	33	NS	NS	NS	Adalimumab
Tonelli et al. 2012 [13] (Ita)	Pilot/uncontrolled study	12	43.5 (27–59)	3:9	7 TS 3 AV 2 ‘complex’	Adalimumab
		92				

*NS* not specified, *TS* transsphincteric, *IS* intersphincteric, *SS* suprasphincteric, *AV* anovaginal, *LA* local anaesthetic

**Table 2:** Treatment regimen

Study	Numbers	Median follow-up in months (range)	Dose (mg)	No. of treatments	Dosing interval (weeks)	Type of LA treatment	Mode of injection
Lichtiger S. 2001 [9] (USA)	9	1	20	3	1 and 2	Infliximab	Circumferential + intrafistula
Poggioli et al. 2005 [12] (Ita)	15	18.2 (3–30)	15–21	≥ 6	4	Infliximab	(Fistulectomy) + Circumferential (IO/EO) + intrafistula
Asteria et al. 2006 [11] (Ita)	11	10.5 (7–18)	20	≥ 3	4	Infliximab	Circumferential (IO/EO) + intrafistula
Alessandroni et al. 2011 [10] (Ita)	12	35 (19–43)	20–25	≥ 2	4–6	Infliximab	Circumferential (IO/EO) + intrafistula
Laureti et al. 2012 [14] (Ita)	33	11^a^ (7–14)	40	≥ 2	2	Adalimumab	Submucosal around I.O.
Tonelli et al. 2012 [13] (Ita)	12	17.5 (5–30)	20	≥ 4	2	Adalimumab	Circumferential (IO/EO) + intrafistula

delete row if possible otherwise, just text please

*IO* internal opening, *EO* external opening, *LA* local anaesthetic

Outcome measures were mostly clinical, i.e. with primary end points being complete or improved healing of fistula. Some studies used radiological techniques (ultrasonography/MRI), in addition to clinical findings [10, 13]. Success rates, as defined by the studies, were signified by complete/partial response to anti-TNF treatment. This was ascertained by clinical examination, to assess for discharge, with complete response signifying absence of discharge/clinical healing. The study with the longest follow-up demonstrated response rates of 62.5%, i.e. 5/8 patients with complete healing (clinical assessment) at median follow-up of 35 months [10]. The response rates in the rest of the studies revealed a partial/complete response varying from 40 to 100% (Table 3). Morbidity was low with the procedure. Reports of minor symptoms of local irritation/burning/heaviness were largely self-limiting. Poggioli and colleagues reported three adverse events in their study of 15 patients—one case of pre-existing rectal stenosis worsened after treatment, one case of new recto-urethral fistula requiring surgery and one case of poor sphincter function after treatment. Alessandroni et al [10] reported a delayed hypersensitivity reaction in a patient who was treated with local infliximab but then subsequently had to abandon treatment and go on to intravenous infliximab due to relapse of intestinal symptoms. The patient developed a delayed hypersensitivity reaction after first infusion and was subsequently lost to follow-up.

**Table 3:** Outcomes

Study	Numbers	Median follow-up in months (range)	Outcomes
Lichtiger S. 2001 [9] (USA)	9	1	44% (4/9) demonstrated complete and 33% (3/9) partial response
Poggioli et al. 2005 [12] (Ita)	15	18.2 (3–30)	67% (10/15) demonstrated complete response
Asteria et al. 2006 [11] (Ita)	11	10.5 (7–18)	36% (4/11) demonstrated complete and 36% (4/11) partial response
Alessandroni et al. 2011 [10] (Ita)	12	35 (19–43)	62.5% (5/8) demonstrated complete response
Laureti et al. 2012 [14] (Ita)	33	11 (7–14)	40% demonstrated complete response
Tonelli et al. 2012 [13] (Ita)	12	17.5 (5–30)	75% (9/12) demonstrated complete and 25%(3/12) partial response

## Discussion

### Local anti-TNF therapy as a potential therapeutic option

The introduction of anti-TNF-α heralded a significant addition to treatment of perianal Crohn’s fistulas. Initial remission rates have been reported as up to 55% in the literature, with maintenance treatment resulting in continued remission in about a third of patients at 1 year [6, 15–18]. Infliximab was the first of the anti-TNF therapies to have demonstrated benefit. Fistula response in the ACCENT 2 trial was prolonged by maintenance intravenous infusion every 8 weeks [16, 19]. This treatment has since been accepted into guidelines in managing fistulising perianal Crohn’s disease [20]. However, as with all immunomodulators, there are risks of adverse events with continued use (e.g. infusion reactions, neurological events, infections). These concerns led to the proposal of local injection of anti-TNF as an alternative to systemic infusion. Theoretical advantages include more efficient delivery with direct diffusion/interstitial fluid movement of antibody to target site, preventing need for high systemic concentrations [21]. Lichtiger initially described the technique in a small case series with injection into the fistula tract and circumferentially around tract (subcutaneously). They reported partial clinical response in 78% (7/9patients) and complete closure in 44% (4/9patients) and no significant adverse events. However, follow-up period was only 1 month, which makes the actual efficacy/healing rates difficult to assess in this study. In our review, three other case series assessed local infliximab injection [10–12]. They used a modified version of the technique (described by Lichtiger) with injection around the internal opening, as well as the fistula tract/external opening; this was combined with debridement/fistulectomy of the tract. Partial/complete response was demonstrated in approximately 62.5–73% at ≥ 1 year. Similarly, no significant adverse events were reported. Dosing (15–25 mg) and intervals (4–6 weeks) were similar.

Two studies have been reported in the literature regarding adalimumab use. Tonelli and colleagues [13] reported a 100% partial/complete response in 12 patients who had 20 mg twice weekly after median follow-up of 18 months. Seventy-five per cent of these demonstrated complete closure. Laureti et al. [14], who followed on from their experience with infliximab [12] reported 40% complete closure (13/33 patients) at 11 months after median number of nine treatments with 40 mg adalimumab.

### Adverse events/limitations of local anti-TNF therapy

In general, the studies on local anti-TNF injections were free of significant adverse events. However, it is important to note the report of delayed hypersensitivity reaction in a patient undergoing intravenous infliximab following previous local therapy. The variable dosing intervals may indeed theoretically provoke such a response and this may be a significant concern with a non-standardised dosing regimen. It is possible that the local injections may stimulate formation of antibodies which, in turn, may render the patient sensitised and intolerant to future systemic treatment [22].

A major limitation in the studies we reviewed is the measurement of response to treatment, i.e. nature of outcome reporting. There is significant heterogeneity of outcome reporting, which represents a widespread issue in assessing outcome of interventions in the perianal Crohn’s fistula literature. The most widely used instrument for assessing treatment outcomes in perianal Crohn’s clinical trials is the fistula drainage assessment [16]. Fistulas are classified as open (i.e. purulent material is expelled with gentle pressure) or closed. A fistula should remain closed for two consecutive visits (at least 4 weeks apart) to be considered closed (complete healing). If half of all external openings are closed, the patient has responded (partial healing). Other studies report fistula drainage ‘semi-quantitatively’ based on frequency/quantity of dressing/pad changes. In our review, most studies reported clinical assessment of healing and classified this as complete or partial (definitions of these were heterogeneous). These clinical outcome measures are subjective and do not account for temporal changes in fistula drainage, and may be subject to recall bias. Clinical outcome reporting is also exacerbated by the fact that clinical healing is not always readily achievable and does not always correlate with radiological healing. In fact, MRI confirmation of deep healing has been shown to occur in a median of 12 months after closure of the external openings [4, 23]. Two studies [10, 13] in our review used radiological outcomes (MRI/endoanal ultrasound) in addition to clinical assessment. In the study by Alessandroni et al. [10], all patients underwent MRI at 1 year follow-up to check complete resolution of fistula/healing, as well as to assess the fistula anatomy, if recurrence was suspected. Interestingly, the MRI was diagnostic of an intersphincteric fistula in a patient that had been clinically deemed to have complete resolution after treatment. Otherwise, the MRI and clinical findings correlated. Tonelli et al. [13] used MRI/USS examination to confirm complete closure of fistula tract and again imaging demonstrated persistence of a fistula in a patient that was deemed to have clinically healed. This required further treatment. Another limitation was the inability to fully stratify according to the type of fistula in other to correlate this with response. Table 2 demonstrates that most fistulas were transsphinteric, and the few numbers of the rest (e.g. intersphincteric/suprasphincteric) make it difficult to accurately compare this.

### Conclusion

In summary, the data available on this technique is significantly limited; outcomes are heterogeneous and make interpretation difficult. There does seem to be a positive response towards fistula healing; however, this is difficult to quantify statistically, given the scant evidence available. Other limitations include the lack of comparison with placebo and furthermore, the heterogeneity and paucity of long-term data with reproducible outcomes make it difficult to determine the duration of remission/recurrence rates. The ideal dosing regimen and intervals remain unclear, as well as the risk of antibody formation and thus hypersensitivity with future treatments. There may however be scope for treatment with local injection of anti-TNF in a subset of patients who are either intolerant to, or in whom systemic therapy is contraindicated.
